# Glu289 residue in the pore-forming motif of *Vibrio cholerae* cytolysin is important for efficient β-barrel pore formation

**DOI:** 10.1016/j.jbc.2022.102441

**Published:** 2022-08-31

**Authors:** Anish Kumar Mondal, Nayanika Sengupta, Mahendra Singh, Rupam Biswas, Kusum Lata, Indrajit Lahiri, Somnath Dutta, Kausik Chattopadhyay

**Affiliations:** 1 Department of Biological Sciences, Indian Institute of Science Education and Research Mohali, Manauli, Punjab, India; 2 Molecular Biophysics Unit, Indian Institute of Science, Bangalore, India

**Keywords:** bacterial toxin, pore-forming toxin, *Vibrio cholerae* cytolysin, membrane, transmembrane domain, oligomerization, membrane pore, prepore, protein structure, cryo-EM, PFT, Pore-forming toxin, SPR, surface plasmon resonance, TEM, transmission electron microscopy, VCC, Vibrio cholerae cytolysin

## Abstract

*Vibrio cholerae* cytolysin (VCC) is a potent membrane-damaging β-barrel pore-forming toxin. Upon binding to the target membranes, VCC monomers first assemble into oligomeric prepore intermediates and subsequently transform into transmembrane β-barrel pores. VCC harbors a designated pore-forming motif, which, during oligomeric pore formation, inserts into the membrane and generates a transmembrane β-barrel scaffold. It remains an enigma how the molecular architecture of the pore-forming motif regulates the VCC pore-formation mechanism. Here, we show that a specific pore-forming motif residue, E289, plays crucial regulatory roles in the pore-formation mechanism of VCC. We find that the mutation of E289A drastically compromises pore-forming activity, without affecting the structural integrity and membrane-binding potential of the toxin monomers. Although our single-particle cryo-EM analysis reveals WT-like oligomeric β-barrel pore formation by E289A-VCC in the membrane, we demonstrate that the mutant shows severely delayed kinetics in terms of pore-forming ability that can be rescued with elevated temperature conditions. We find that the pore-formation efficacy of E289A-VCC appears to be more profoundly dependent on temperature than that of the WT toxin. Our results suggest that the E289A mutation traps membrane-bound toxin molecules in the prepore-like intermediate state that is hindered from converting into the functional β-barrel pores by a large energy barrier, thus highlighting the importance of this residue for the pore-formation mechanism of VCC.

Pore-forming toxins (PFTs) are a unique class of protein toxins with potent cell-killing activity. PFTs exert their toxic effects by forming pores in the lipid bilayer of the plasma membrane. Pore formation causes severe damage to the plasma membrane that can eventually lead to the death of the target cells (1). PFTs are produced by a wide variety of organisms, and they play important roles in diverse biological processes. In particular, many pathogenic bacteria employ PFTs as their virulence factors (2). Based on the structural signature of the pore-forming motifs, PFTs are generally classified into two distinct categories: (i) α-PFTs that employ α-helical bundles to form membrane-inserted pores and (ii) β-PFTs that utilize β-barrels to generate transmembrane pores (3).

*Vibrio cholerae* cytolysin (VCC) is a distinct member in the β-PFT family (4, 5). VCC is produced by most of the pathogenic strains of *V. cholerae*, a Gram-negative bacterium that causes severe diarrheal disease cholera (6). VCC is considered to be a potent virulence factor of *V. cholerae*. It exhibits several pathophysiological activities that include hemolytic activity, cytotoxicity, and enterotoxicity (7, 8, 9). VCC is secreted by the bacteria as an inactive precursor Pro-VCC. Upon secretion from the bacterial cells, the N-terminal pro-domain is proteolytically removed by the bacterial proteases, and the mature form of VCC is generated (10, 11). Mature VCC is composed of a central cytolysin domain (Fig. 1*A*) that contains a distinct pore-forming motif constituted from an anti-parallel β-strand pair, designated as the prestem motif (Fig. 1, *A* and *B*). VCC also harbors a β-trefoil lectin-like domain followed by a β-prism lectin-like domain at the C-terminal boundary of the cytolysin domain (Fig. 1*A*) (12).

**Figure 1:**
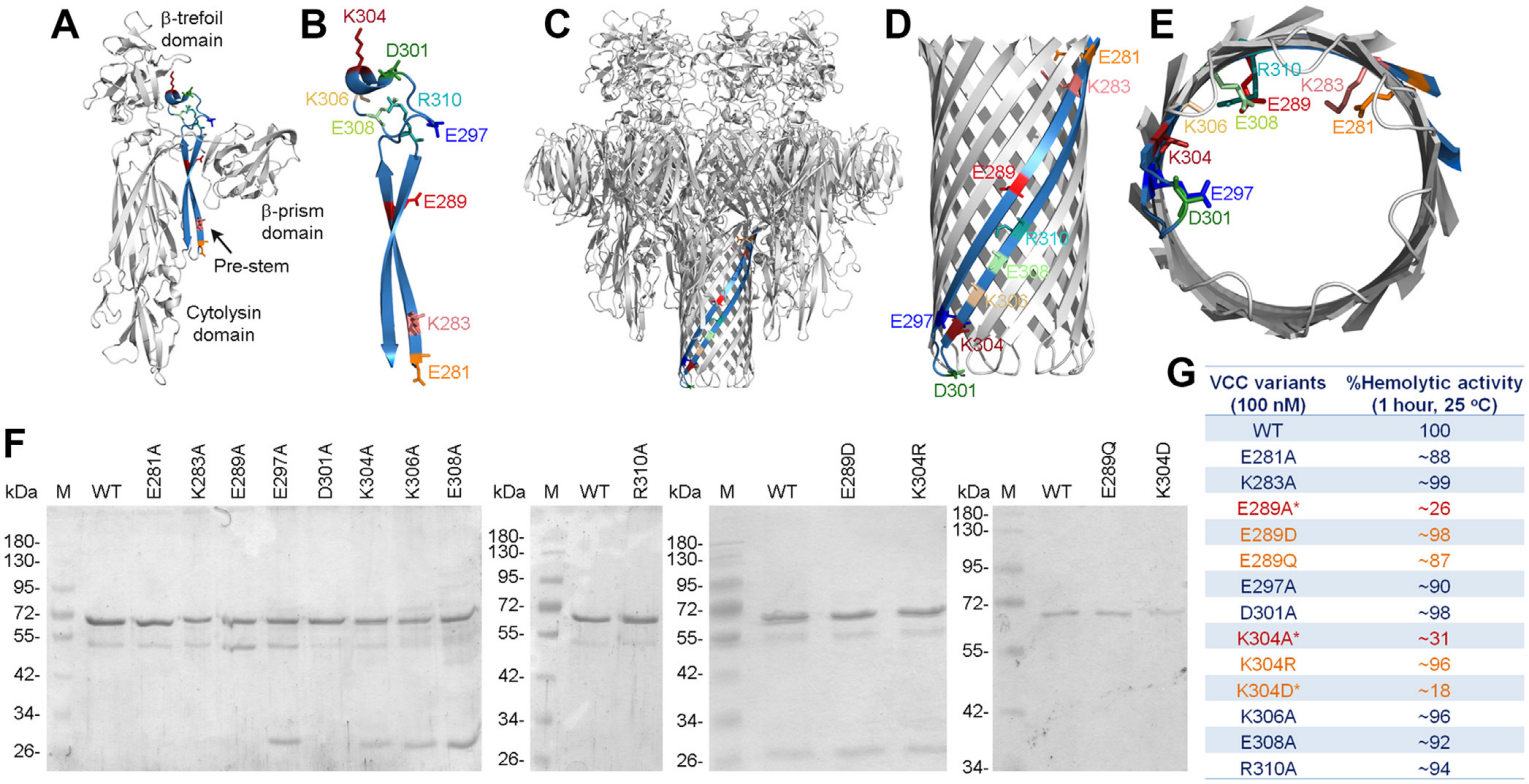
**The pore-forming motif of VCC harbors nine charged residues (E281, K283, E289, E297, D301, K304, K306, E308, and R310) that get positioned toward the lumen of the β-barrel pore upon oligomeric pore-formation.***A*, structural model of the monomeric state of VCC (generated based on the PBD ID 1XEZ). The pore-forming motif in its prestem configuration is shown in *blue color*. The nine specific charged residues within the prestem are indicated in different colors. *B*, zoomed view of the pore-forming prestem motif of VCC shows the locations of the nine charged residues. *C*, structural model of the heptameric β-barrel pore of VCC (generated based on the PBD ID 3O44). For simplicity, the pore-forming motif of only one protomer is shown in *blue color*. The specific nine charged residues are highlighted with different colors. *D*, zoomed view of the β-barrel scaffold of the VCC pore is shown. *E*, the bottom-up view of the transmembrane β-barrel scaffold of VCC shows the locations of the nine specific charged residues within the water-filled lumen of the pore. *F*, SDS-PAGE/Coomassie staining profile of the purified VCC variants (WT and the mutants). Lanes M, molecular weight markers. *G*, pore-forming hemolytic activity of the VCC variants (WT and the mutants) against the human erythrocytes. Hemolytic activity was estimated by monitoring the decrease in the turbidity of the erythrocyte suspension (corresponding to the absorbance at 650 nm). The two mutants, E289A and K304A, displaying the compromised hemolytic activity, are highlighted in *red color* and marked with an *asterisk*. VCC, *Vibrio cholerae* cytolysin.

VCC exists as monomers in solution (13). VCC monomers bind to the cell membrane and form transmembrane heptameric β-barrel pores (Fig. 1*C*) (5, 14, 15, 16). Multiple interaction mechanisms promote efficient binding of VCC toward the target membrane (17). VCC employs specific lipid-dependent interactions to bind to the target membrane. Three loop regions at the bottom tip of the cytolysin domain of VCC play a crucial role in binding to the membrane phospholipid headgroups (18). Moreover, an efficient pore-formation process is critically dependent on the interaction of VCC with the membrane cholesterol (19). In addition to the lipid-dependent interactions, a specific lectin-like activity conferred by the β-prism domain also favors VCC binding to the cell-surface glycans present in the target cell membrane (20).

VCC follows the overall mechanism of membrane pore formation employed by the archetypical small pore-forming β-PFTs (21). Soluble toxin monomers bind to the target membrane and form heptameric β-barrel pores (14). Transmembrane β-barrel scaffold is constituted by the pore-forming motifs contributed by each of the seven toxin protomers (5). The process is associated with a major structural rearrangement of the pore-forming motif. In the soluble monomeric state, pore-forming motif remains folded as prestem within the core cytolysin domain (12). During the oligomeric pore formation, prestem motif of each of the toxin protomers detaches from the cytolysin domain and inserts into the membrane lipid bilayer in a concerted manner to form the stem of the transmembrane β-barrel (5). Pore-formation mechanism of the archetypical small pore-forming β-PFTs, in general, proceeds through formation of transient metastable oligomeric intermediates that are typically designated as prepores. The prepore assembly is an intermediate in which the pore-forming motifs of the toxin protomers are proposed to be in a partially collapsed state and are not fully inserted into the membrane lipid bilayer (21, 22, 23). Functional pores are generated only upon complete membrane insertion of the pore-forming motifs creating transmembrane β-barrel. Consistent with such a mechanism of β-barrel pore formation, formation of the prepore intermediate(s) has also been reported for VCC (24).

Pore-forming motif is the most crucial structural feature of any β-PFT. However, pore-forming motifs of the β-PFTs share very little sequence similarity. Nevertheless, they share one common feature. Surface of the transmembrane β-barrel that faces the hydrophobic interior of the membrane lipid bilayer is populated with hydrophobic residues. In contrast, pore lumen is lined primarily by the polar and charged residues (25). Such a remarkable structural feature allows the pore-forming motifs of the β-PFTs to form water-filled pores within the hydrophobic interior of the membrane lipid bilayer. Amino acid composition of the pore-forming motif appears to play a major role in dictating its overall structural disposition during the mode of action of a β-PFT. Also, some of the residues within the pore-forming motif play more crucial roles than others. In one of our previous studies, we have examined the role of the four specific aromatic residues of the prestem motif of VCC (26). In the water-soluble monomeric state, these residues remain buried against the cytolysin domain. In the oligomeric pore form, they are protruded outwardly from the β-barrel stem. Out of these four aromatic residues, only the tyrosine residue at position 321 (Y321) is found to be essential for the pore-forming functionality of VCC. Y321 appears to maintain an optimal structural constraint at the hinge region of prestem that plays a crucial role in regulating the structural rearrangement of the pore-forming motif during oligomeric pore formation (26).

Consistent with the archetypical design of the β-PFTs, VCC prestem harbors a series of charged residues. Nine out of these charged residues get positioned into the water-filled lumen of the β-barrel pore. These nine residues are as follows: (1) glutamate at position 281 (E281), (2) lysine at position 283 (K283), (3) glutamate at position 289 (E289), (4) glutamate at position 297 (E297), (5) aspartate at position 301 (D301), (6) lysine at position 304 (K304), (7) lysine at position 306 (K306), (8) glutamate at position 308 (E308), and (9) arginine at position 310 (R310) (Fig. 1, *A*–*E*). In the present study, we explore the implications of these prestem residues for the β-barrel pore-formation mechanism of VCC.

## Results

### Mutations of E289A and K304A within the prestem motif compromise membrane-damaging pore-forming activity of VCC

In the present study, we examined the implications of the prestem charged residues for the pore-forming functionality of VCC. For this, we targeted nine specific residues that face the pore lumen interior in the oligomeric pore state (Fig. 1*E*). We generated mutant variants of VCC; in each of these, one of the target residues was replaced with alanine (Fig. 1*F*). VCC mutants (E281A, K283A, E289A, E297A, D301A, K304A, K306A, E308A, and R310A) were tested for their pore-forming hemolytic activity against the biomembranes of human erythrocytes, using WT VCC as control (Fig. 1*G*). For some of the residues (such as E289 and K304; alanine replacement of which caused compromised hemolytic activity, as discussed below), additional mutations (E289D, E289Q, K304R, and K304D) were also introduced to examine the effects on the hemolytic activity (Fig. 1, *F* and *G*).

**Figure 2:**
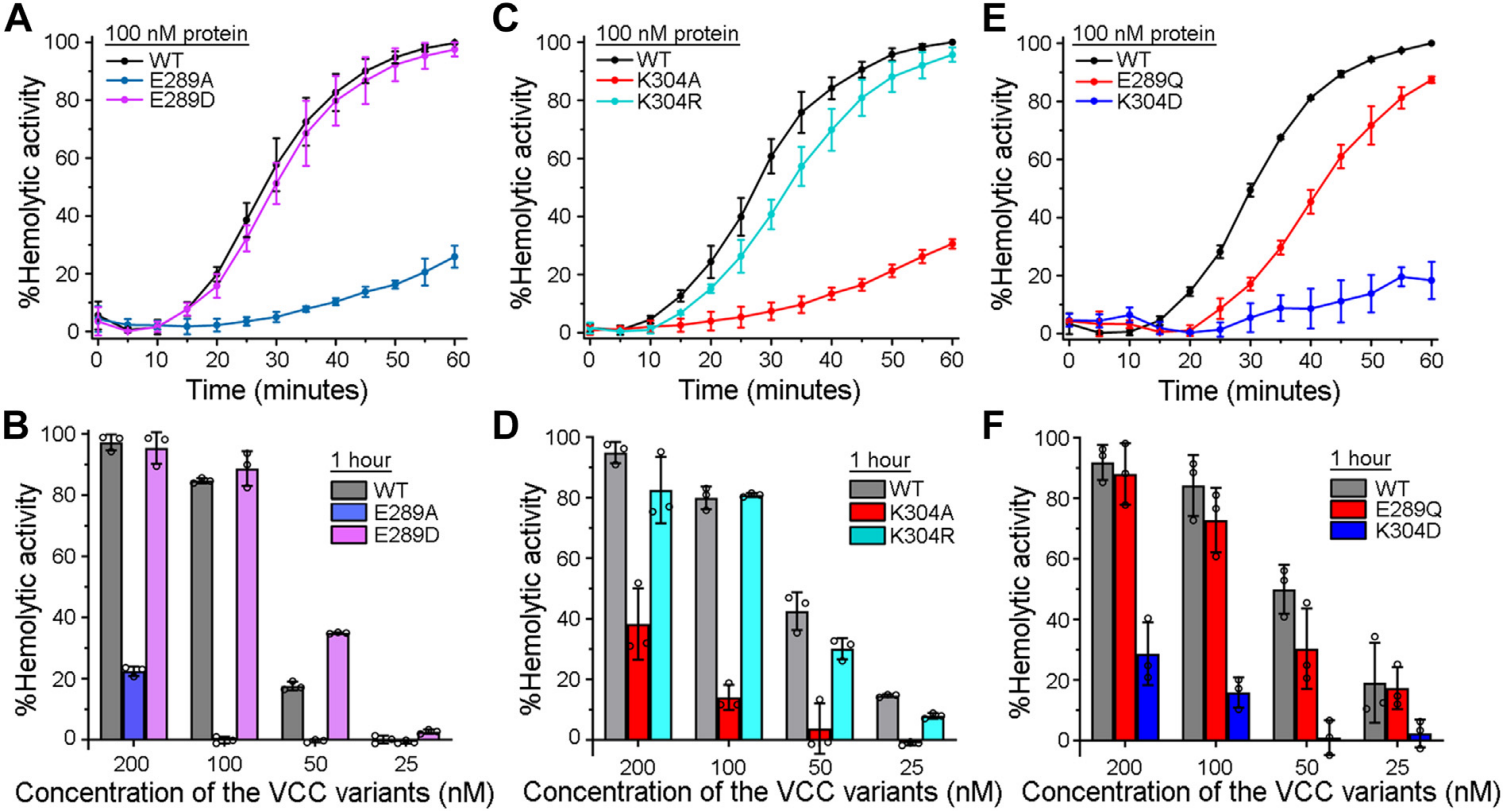
**Mutations of E289A and K304A in the pore-forming motif of VCC compromise the hemolytic activity against human erythrocytes.***A*, *C*, and *E*, pore-forming hemolytic activity of the VCC variants (WT and mutants; 100 nM) was monitored for 1 h at 25 °C by measuring the decrease in the turbidity of the erythrocyte suspension (corresponding to the absorbance at 650 nm). *B*, *D*, and *F*, hemolytic activity of the VCC variants (WT and mutants; at four different protein concentrations) was estimated by monitoring the release of hemoglobin (by measuring absorbance at 415 nm) upon lysis of the human erythrocytes at 25 °C. Data shown here are the averages ± SDs from the three independent treatments. VCC, *Vibrio cholerae* cytolysin.

Hemolytic activity was estimated by monitoring decrease in the turbidity (corresponding to the absorbance at 650 nm) of the erythrocyte suspension due to the cell-lysis over a period of 1 h upon treatment with 100 nM protein, at 25 °C. Under this experimental condition, mutations of E289A and K304A in VCC resulted in a drastic reduction of the hemolytic activity (Figs. 1*G,* 2, *A* and *C*), compared to that of WT-VCC. Both the mutants, E289A-VCC and K304A-VCC, showed ∼75 to 70% reductions in their hemolytic activity with respect to that of the WT protein. Alanine mutations of the other target residues did not affect the hemolytic activity to any noticeable extent (Fig. 1*G*). Interestingly, mutations of E289D and K304R did not affect the hemolytic activity to any noticeable extent (Figs. 1*G* and 2). E289Q mutant also displayed hemolytic activity nearly similar to that observed with WT-VCC. Furthermore, the mutation of K304D resulted in a drastic reduction in the hemolytic activity of VCC (Figs. 1*G* and 2*E*).

Compromised hemolytic activity of the E289A and K304A mutants was further confirmed over a range of protein concentrations (25, 50, 100, and 200 nM) (Fig. 2, *B* and *D*). In this assay of hemolytic activity, the extent of hemoglobin release from the lysed erythrocytes was measured upon treatment with the VCC variants (at varying concentrations) for a period of 1 h, at 25 °C. The result of this assay confirmed that at all the concentrations used, both E289A and K304A mutants showed drastically reduced hemolytic activity as compared to WT-VCC. Even at the concentration of 200 nM, E289A displayed a nearly 80% reduction in the activity, while K304A showed a nearly 60% reduction in the hemolytic activity, compared to ∼100% activity of the WT protein. The mutants, E289D, E289Q, and K304R, displayed nearly WT-like hemolytic activity at all the concentrations (Fig. 2, *B*, *D*, and *F*). In contrast, K304D mutant showed severely compromised activity at all the protein concentrations tested (Fig. 2*F*).

Altogether, these results confirmed that the presence of a specific set of residues at the sites 289 (E, D, or Q) and 304 (K or R), within the pore-forming prestem motif of VCC, is vital for the membrane-damaging pore-forming activity of the toxin.

### K304A mutant shows altered tertiary structural integrity, while E289A mutant is having WT-like structural feature

**Figure 3:**
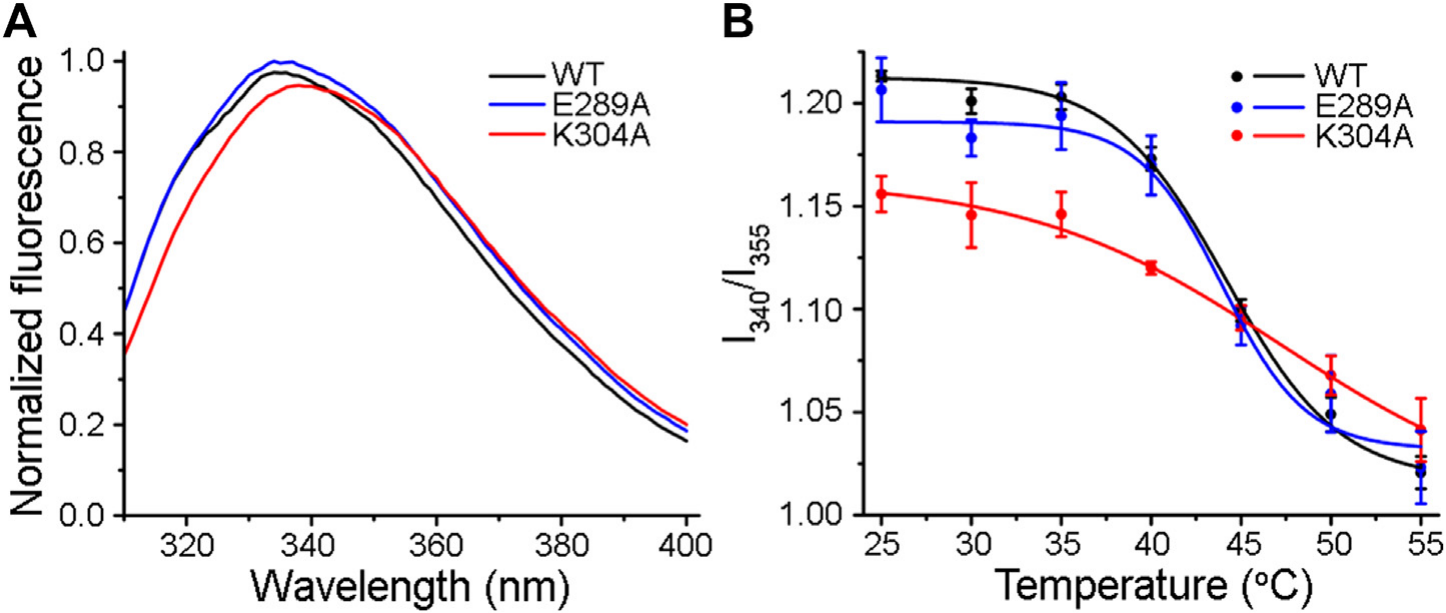
**Intrinsic tryptophan fluorescence emission profile of the VCC variants.***A*, intrinsic tryptophan fluorescence emission spectra of the VCC variants. Fluorescence emission spectra were normalized with respect to the maximum fluorescence intensity value among all the three spectra. *B*, thermal unfolding profiles of the VCC variants were obtained from the tryptophan fluorescence emission spectra collected at various temperatures. The ratio of the fluorescence emission intensities at 340 nm and 355 nm (I_340_/I_355_) was plotted against the temperature to monitor the overall tertiary structural changes upon the thermal unfolding of the VCC variants. Data represent averages ± SDs from three independent experiments. VCC, *Vibrio cholerae* cytolysin.

The mature form of WT-VCC has 11 tryptophan residues distributed throughout its structure. Therefore, intrinsic tryptophan fluorescence emission profile of VCC serves as a distinct indicator of its overall tertiary structural disposition. The E289A mutant showed an intrinsic tryptophan fluorescence emission spectrum almost overlapping with that of WT-VCC. In contrast, the K304A mutant displayed a red-shifted tryptophan fluorescence emission profile (Fig. 3*A*). This observation suggested that the mutation of K304A resulted in a noticeable alteration in the protein’s overall tertiary structure compared to WT-VCC.

Analyses of the intrinsic tryptophan fluorescence emission (ratio of fluorescence intensities at 340 nm and 355 nm) over a range of temperatures (25–55 °C) revealed a distinct thermal unfolding profile for the K304A mutant, as compared to that of WT-VCC. In contrast, the E289A mutant showed a thermal unfolding profile that was nearly similar to that of the WT protein (Fig. 3*B*).

Overall, these data suggested that the mutation of K304A, but not E289A mutation, altered the overall structural integrity of VCC in its water-soluble monomeric state.

### E289A mutant retains ability to associate with the target membranes and form oligomeric assembly similar to that observed with WT VCC

#### Binding and oligomerization in the membrane lipid bilayer of cholesterol-containing liposomes

**Figure 4:**
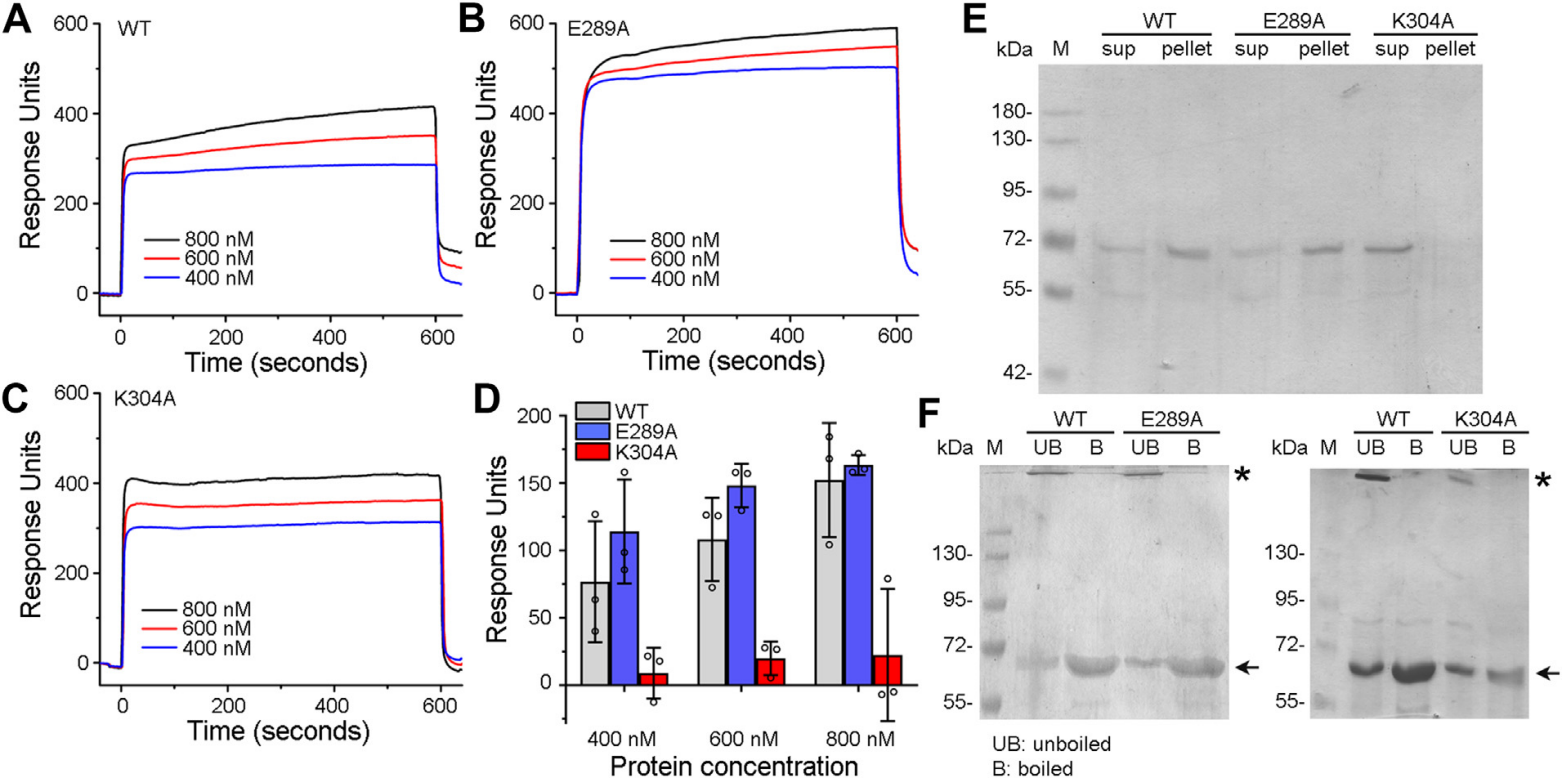
**Binding and SDS-stable oligomer formation by the VCC variants in the Asolectin-cholesterol liposomes.***A*–*C*, real-time SPR sensograms show the binding of the VCC variants (WT, E289A, and K304A) with the membrane lipid bilayer of the Asolectin-cholesterol liposomes. Three different protein concentrations (400, 600, and 800 nM) were used. Proteins were injected over the liposome-coated SPR sensor chips for 600 s. Subsequently, the buffer was injected to wash off the nonspecifically bound proteins to monitor the end-point response corresponding to the irreversibly bound proteins. *D*, the end-point response obtained from the SPR-based binding assay shows the irreversible binding of the VCC variants with the liposome membranes. Data shown here are the averages ± SDs from the three independent experiments. *E*, pull down–based assay to monitor the binding efficacies of the VCC variants (WT, E289A, and K304A; 1 μM) with the Asolectin-cholesterol liposomes. Pellet fractions represent the liposome-bound proteins, while the supernatant (Sup) fractions indicate the unbound proteins. Fractions were analyzed by SDS-PAGE/Coomassie staining. *F*, SDS-stable oligomer formation by the VCC variants (1 μM) in the Asolectin-cholesterol liposome membranes. Membrane-bound pellet fractions were analyzed by SDS-PAGE/Coomassie staining with or without boiling in the presence of the SDS-PAGE sample buffer. Bands corresponding to the SDS-stable oligomers are indicated with *asterisks*, and the monomeric bands are indicated with an *arrow*. VCC, *Vibrio cholerae* cytolysin; SPR, surface plasmon resonance.

Toward exploring the mechanistic basis of compromised pore-forming ability of the E289A mutant, we first examined its binding ability toward the membrane lipid bilayer of Asolectin-cholesterol liposomes. In the surface plasmon resonance (SPR)–based assay of binding, E289A-VCC (at three different concentrations; 400 nM, 600 nM, and 800 nM) showed efficient interaction with the membrane lipid bilayer of Asolectin-cholesterol liposomes, similar to that observed with WT-VCC (Fig. 4, *A*–*D*). Analyses of the steady-state binding profiles (as observed from the real-time SPR sensogram)(Fig. 4*B*), as well as quantitation of the irreversible binding (based on the end-point response; at the end of protein injection and during buffer wash)(Fig. 4*D*), suggested that E289A-VCC could efficiently associate with the liposomes and could irreversibly bind to the membrane lipid bilayer.

Pull down–based assay of binding also confirmed that the E289A mutant could efficiently associate with the Asolectin-cholesterol liposomes (Fig. 4*E*), as observed with WT-VCC. In this assay, VCC variants (1 μM) were incubated with Asolectin-cholesterol liposomes. Subsequently, the reaction mixtures were subjected to ultracentrifugation to collect the pellet and supernatant fractions for probing liposome-bound protein and unbound protein, respectively. Result of this assay clearly showed that E289A-VCC was enriched in the liposome-bound pellet fraction, similar to WT-VCC (Fig. 4*E*).

We examined whether E289A mutant could form oligomeric assembly after establishing an efficient interaction with the liposome membrane. Archetypical β-PFTs, including VCC, form SDS-stable oligomers in the membrane lipid bilayer. Such SDS-stable oligomers can be detected on SDS-PAGE when probed under the unboiled condition in the presence of SDS-PAGE sample buffer. Based on such notion, in the present study, VCC variants (1 μM) were incubated with Asolectin-cholesterol liposomes, and liposome-bound pellet fractions were probed for SDS-stable oligomer formation. Our result showed that the membrane-bound fraction of E289A-VCC formed SDS-stable oligomers, similar to that formed by WT-VCC (Fig. 4*F*). Therefore, these results suggested that the mutation of E289A in VCC did not affect binding and oligomerization ability in the membrane lipid bilayer of liposomes to any noticeable extent.

#### Binding and oligomerization in the biomembranes of human erythrocytes

**Figure 5:**
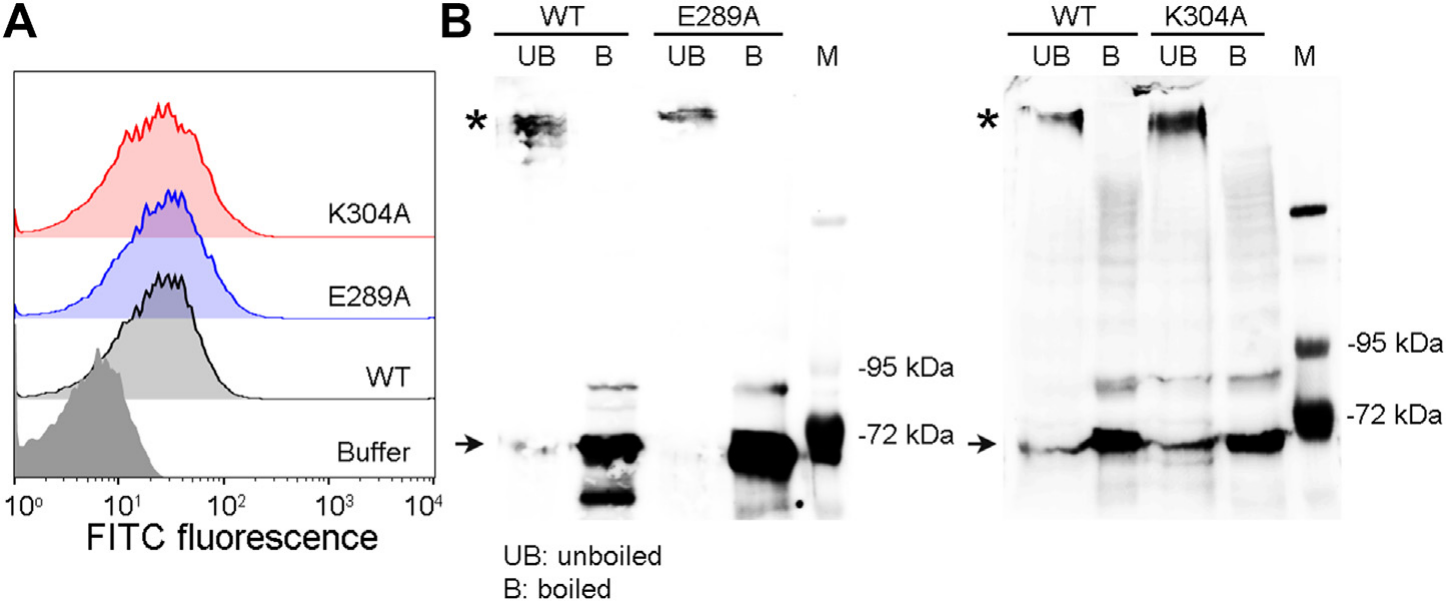
**Binding and SDS-stable oligomer formation by the VCC variants in the biomembranes of human erythrocytes.***A*, the binding efficacy of the VCC variants (WT, E289A, and K304A; 75 nM) with the human erythrocytes was monitored by a flow cytometry–based assay. *B*, pull down–based assay was used to monitor SDS-stable oligomer formation by the VCC variants (100 nM) in the human erythrocytes membrane ghosts. Membrane-bound fractions were analyzed by SDS-PAGE, with or without boiling in the presence of the SDS-PAGE sample buffer, followed by immunoblotting with the anti-VCC antibody. Lanes M show the molecular weight markers. Bands corresponding to the SDS-stable oligomers are indicated with *asterisks*, and the monomeric bands are indicated with an *arrow*. VCC, *Vibrio cholerae* cytolysin.

Toward exploring further the effect of E289A mutation on the pore-formation mechanism of VCC, we examined binding and oligomerization ability of the mutant in the biomembranes of erythrocytes. For this, we first employed a flow cytometry–based assay to monitor the binding of the VCC variants with erythrocytes’ cell surface. In this assay, human erythrocytes were treated with the VCC variants (75 nM) at a lower temperature of 4 °C. Incubation at such low temperature allows VCC binding to erythrocytes but blocks oligomeric pore formation by the membrane-bound toxin and thus prevents lysis of the cells. Therefore, cell populations having bound VCC variants can be probed by flow cytometry upon staining with the anti-VCC antibody. Flow cytometry–based assay showed that E289A-VCC bound to the cell surface of human erythrocytes, similar to that observed with WT-VCC (Fig. 5*A*).

Next, we examined SDS-stable oligomer formation by the E289A mutant upon binding with erythrocytes’ membrane ghost. For this, human erythrocytes’ membrane ghosts were incubated with the VCC variants (100 nM), and membrane-bound fractions were pelleted by ultracentrifugation and probed by SDS-PAGE and immunoblotting with anti-VCC. Membrane-bound fractions of E289A mutant showed SDS-stable oligomer bands when examined under the unboiled condition in the presence of SDS-PAGE sample buffer (Fig. 5*B*).

Altogether, these results showed that E289A-VCC was capable of binding with the biomembranes of human erythrocytes and were able to form SDS-stable oligomeric assembly, similar to those observed with the WT toxin.

#### Transmission electron microscopy to probe ring-like assembly state in the target membranes

**Figure 6:**
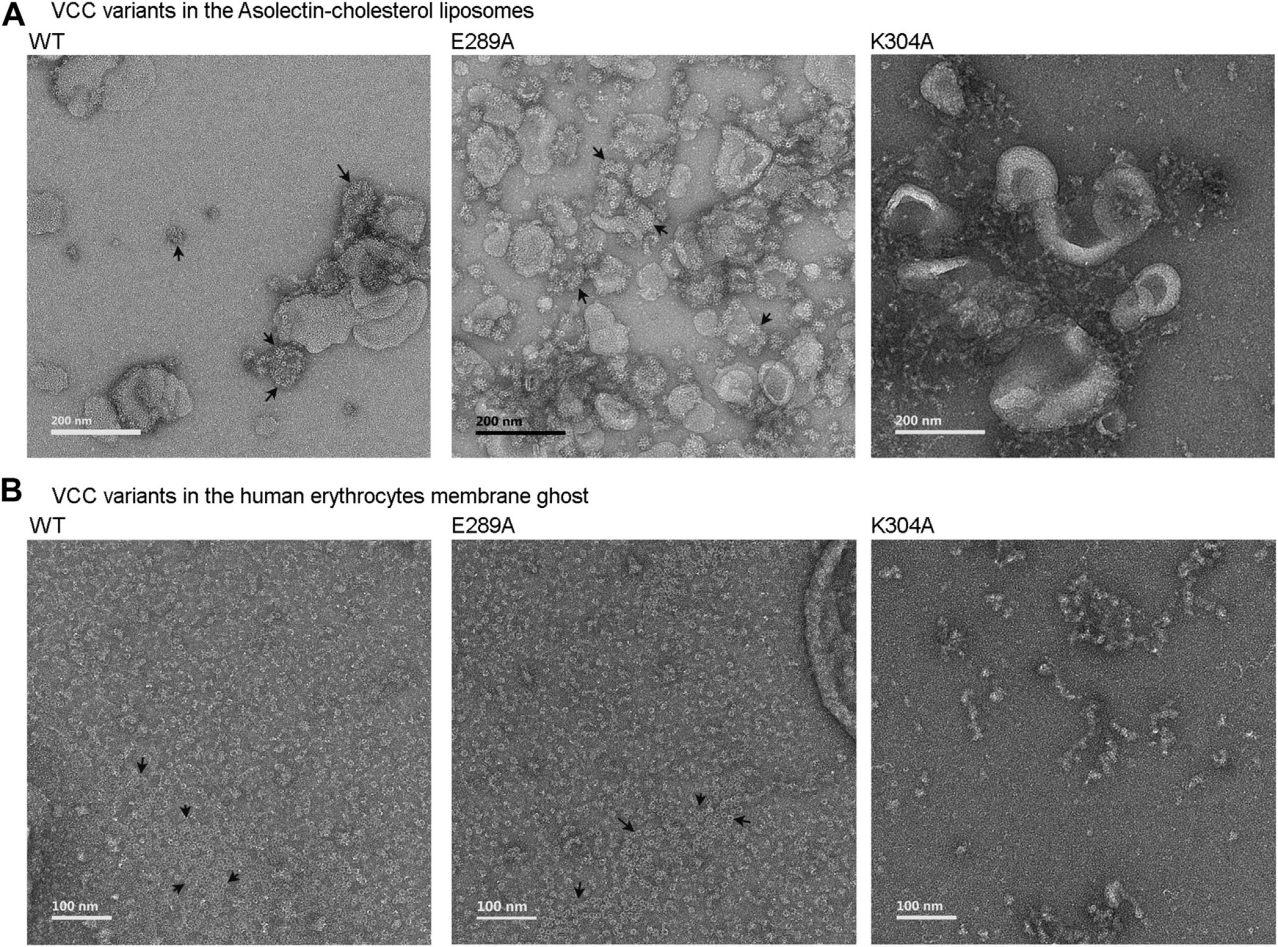
**Transmission electron micrographs showing the assembly state of the VCC variants upon association with the membrane lipid bilayer of the Asolectin-cholesterol liposomes and human erythrocytes membrane ghosts.***A*, assembly states of the VCC variants in the membrane lipid bilayer of the Asolectin-cholesterol liposomes. *B*, assembly states of the VCC variants in the biomembranes of human erythrocytes ghosts. Ring-like oligomeric structures of the VCC variants are indicated with *arrows*. VCC, *Vibrio cholerae* cytolysin.

Transmission electron microscopy (TEM)–based imaging allows visualization of the characteristic ring-like structures of the membrane-bound oligomeric pores formed by the β-PFTs (27). Therefore, we employed negative stain TEM to examine the assembly state of E289A-VCC in the membrane lipid bilayer of Asolectin-cholesterol liposomes and in the biomembranes of human erythrocytes’ ghosts. Consistent with the earlier reports (26), we observed typical ring-like structures formed by WT-VCC upon association with the Asolectin-cholesterol liposomes and human erythrocytes’ membrane ghosts (Fig. 6, *A* and *B*). E289A-VCC also formed similar ring-like assemblies in the liposomes and in the membrane ghosts of human erythrocytes (Fig. 6).

Taken together, our data showed that the E289A mutant, although having compromised pore-forming activity, retained the ability to associate with the target membranes and form oligomeric assembly with typical ring-like architecture, similar to those observed with WT-VCC.

### Consistent with its altered structural integrity, K304A mutant shows abortive membrane interaction and oligomerization

Toward exploring the mechanistic basis of its compromised pore-forming ability, we examined membrane interaction and oligomerization ability of K304A-VCC. In the SPR-based assay of binding, K304A mutant showed WT-like steady-state association with the liposome membrane (Fig. 4, *A* and *C*). However, it failed to establish efficient irreversible interaction with the membrane lipid bilayer (Fig. 4*D*). Pull down–based assay of binding also revealed compromised interaction of K304A mutant with Asolectin-cholesterol liposomes (Fig. 4*E*). Consistent with its reduced binding efficacy, K304A-VCC also displayed a marked reduction in its ability to form SDS-stable oligomers upon incubation with liposomes (Fig. 4*F*). Interestingly, K304A-VCC displayed efficient binding ability toward erythrocytes, similar to those of E289A mutant and WT-VCC (Fig. 5*A*). It also formed SDS-stable oligomers in the erythrocytes’ membrane ghosts (Fig. 5*B*). Accessory interactions available in the biomembranes of erythrocytes might have facilitated binding and oligomer formation by the K304A mutant. However, we failed to detect ring-like assembly state (observed with WT-VCC as well as E289A mutant) for the membrane-bound fraction of the K304A mutant. Negative stain TEM images showed that the membrane-bound fraction of K304A mutant mostly formed heterogeneous aggregates, both in Asolectin-cholesterol liposomes and in human erythrocytes’ membrane ghost (Fig. 6).

Taken together, our results showed abortive membrane interaction and oligomerization ability of K304A-VCC, presumably due to its altered structural integrity, and thus explain compromised pore-forming activity of this mutant.

### Cryo-EM 3D structures of E289A-VCC oligomeric assembly states in the lipid membrane resemble those of WT toxin

**Figure 7:**
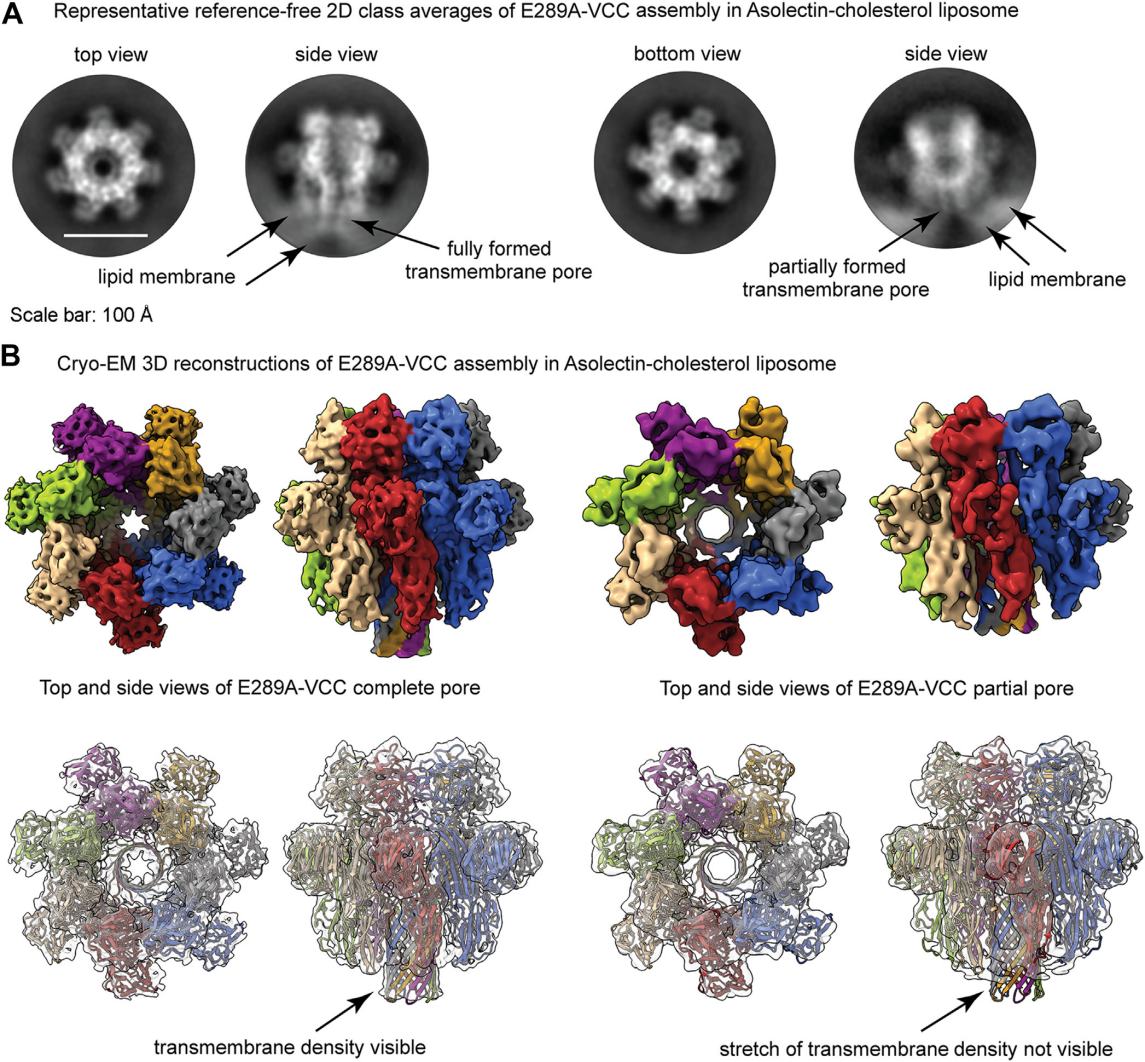
**Cryo-EM 3D reconstruction of liposome-embedded oligomeric assembly of E289A-VCC.***A*, reference-free 2D class averages corresponding to the top and bottom views indicate heptameric ring-like assembly of E289A-VCC in the Asolectin-cholesterol liposome. The membrane-bound side views represent two plausible conformational states of the mutant where different lengths of the transmembrane channel are visible (marked by the *arrows*). *B*, two unique structural states of E289A-VCC, reconstructed through single particle analysis, show different assemblies. Top panel represents the density maps of the E289A mutant, in which complete (EMD-33215) and partially formed transmembrane densities (EMD-33219) are observed. Bottom panel represents the overall fitting of the atomic model of VCC pore structure within the cryo-EM density maps of E289A-VCC. The atomic model docked into the maps with complete and partial transmembrane β-barrel density corresponds to the PDB ID 7YL9. VCC, *Vibrio cholerae* cytolysin.

Our data so far showed that the mutation of E289A within the pore-forming prestem motif of VCC drastically affected pore-forming activity of the toxin, without affecting structural integrity and membrane-binding potential of the mutant. E289A-VCC also formed typical ring-like oligomeric assembly in the target membranes, similar to that formed by WT-VCC. To gain further structural insight into the ring-like oligomeric assemblies formed by E289A-VCC in the target membranes, high resolution cryo-EM was performed to visualize liposome-bound E289A mutant in a near-native environment. As previously observed for the liposome-embedded WT-VCC, E289A oligomers were found to stably associate with the lipid membranes (Fig. S1). The cryo-EM raw micrographs revealed uniformly distributed liposomes studded with ring-like oligomers of E289A mutant. To better evaluate the oligomeric β-PFT pore assembly of E289A-VCC, reference-free 2D class averaging was performed to increase the signal-to-noise ratio of the vitrified protein particles. Cryo-EM 2D class averages indicated the presence of homogeneous oligomeric assembly interacting with the lipid membrane (Figs. 7*A* and S1*B*). Interestingly, the side views of oligomeric E289A-VCC conjugated with lipid membrane showed distinct classes where the extent of transmembrane scaffold engagement with the lipid density appeared to differ. After multiple rounds of initial curation, nearly 234,000 protein particles were subjected to a robust 3D classification step for addressing the apparent heterogeneity in membrane anchoring. Subsequent analysis of the class distribution indicated the presence of oligomeric assemblies that were fully membrane embedded, along with oligomers that appeared to be superficially membrane bound. It is noteworthy, however, to mention that a minor population of possible ‘pores-in-transit’ has been previously reported for WT-VCC (4). In contrast, with the E289A mutant, ∼55% of the oligomers were documented in the states, in which complete transmembrane β-barrel density could not be visualized (Fig. S2). The proportion of the states with complete β-barrel significantly dropped from ∼69% in the case of WT-VCC (as reported previously) (4) to ∼44% for E289A-VCC (as observed in the present study). Thus, while mutation of E289A in the pore-forming motif did not completely abolish pore formation potential, it appeared to dampen the population of such fully bilayer-embedded pore state.

**Figure 8:**
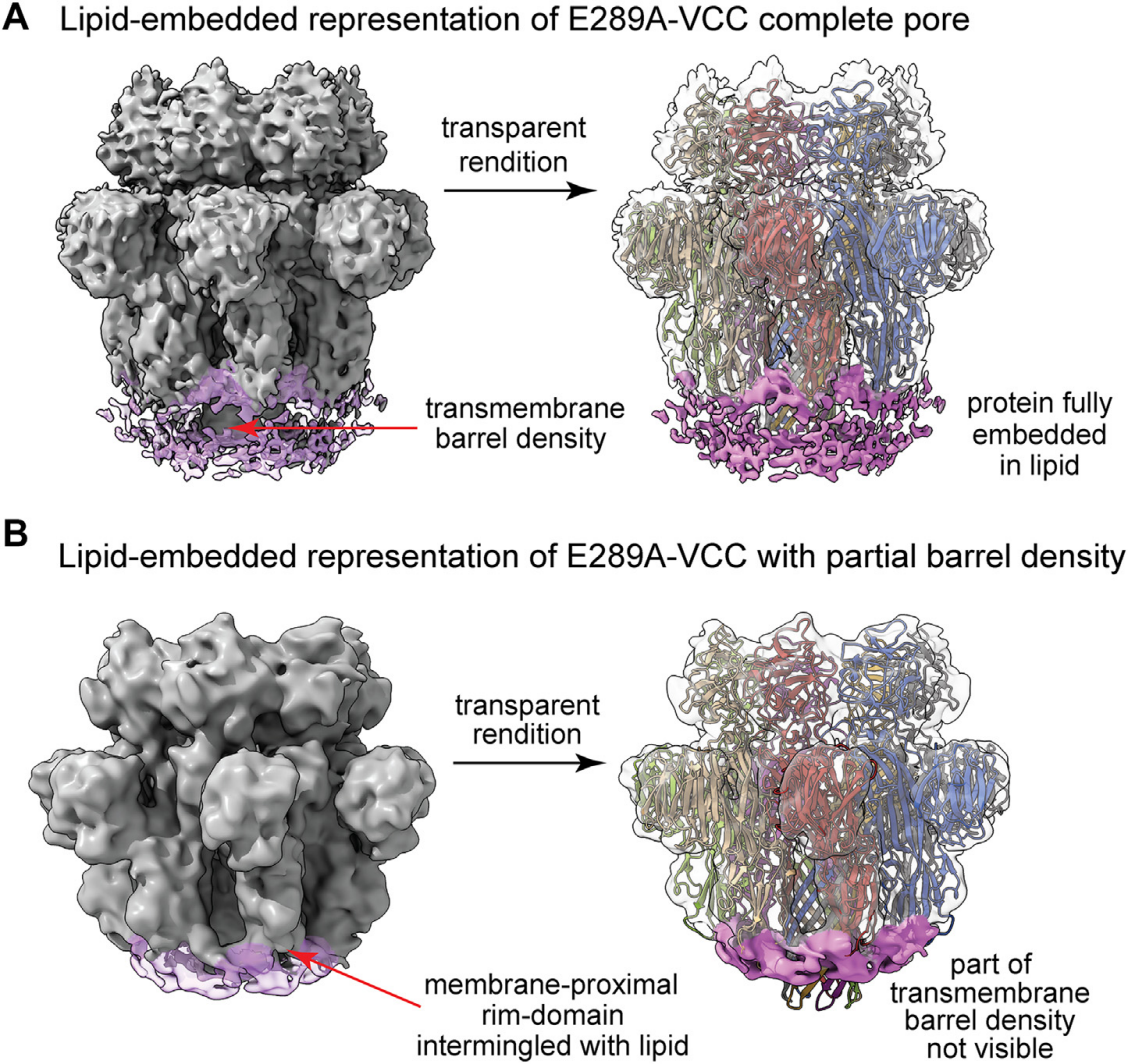
**Membrane-interaction of the assembly states of E289A-VCC.***A*, cryo-EM map of E289A-VCC assembly with full-length β-barrel visualized at lower threshold of density shows the presence of two distinct leaflets of the lipid bilayer. Docking the atomic model (PDB ID 7YL9) shows that the transmembrane pore is fully immersed in the lipid bilayer, corresponding to complete pore-formation. *B*, cryo-EM map of E289A-VCC assembly, in which partial β-barrel could be documented, visualized at lower threshold of density shows the presence of a single layer of lipid density where the rim-domain of the PFT is tightly engaged. Docking the atomic model (PDB ID 7YL9) shows that the length of the density corresponding to the transmembrane β-barrel pore is remarkably shorter in this assembly state of E289A-VCC, thus resembling a probable prepore-like architecture. The lipid densities have been denoted in *pink color*. VCC, *Vibrio cholerae* cytolysin; PFT, pore-forming toxin.

Further analysis of the population distribution of E289A-VCC revealed two discrete states: (i) oligomeric pore, in which full-length transmembrane β-barrel formation was complete and (ii) oligomer, in which partial and distinctly short β-barrel could be visualized (Figs. 7*B* and S2). The structural state resembling WT-VCC pore was resolved at 4.7 Å in a lipid membrane-embedded condition. Analysis of the EM density map indicated clear densities representing the two leaflets of the liposome bilayer (Fig. 8*A*). The overall architecture of the complete pore-forming state of the E289A mutant showed good correlation with the atomic model derived from the crystal structure of the heptameric VCC pore (PDB ID 3O44) (5) (Figs. 7*B* and S3). An observed EMRinger score (28) of 1.07 provided a metric for the quality of the real-space refinement at the global resolution of 4.7 Å (Table S1). Appearance of side chain densities for bulky amino acid residues such as R282 and W318 near the mouth of the barrel also indicated the high resolution of the 3D reconstruction (Fig. S3). Consistent with our expectation, following F288, density for the side chain of E289 was not detected in the alanine-substituted mutant E289A-VCC. However, subsequent densities corresponding to the antiparallel β-strands progressed unhindered to form complete pore in this structural state of E289A-VCC (Fig. S3). The second structural state, in which the density corresponding to the length of the transmembrane β-barrel scaffold was significantly short, was resolved at a moderate resolution of 6.4 Å (Figs. 7*B* and S2). Although the mushroom-head like portion of the structure closely resembled those of WT-VCC pore and complete pore state of E289A-VCC, fitting of the atomic model corresponding to the complete pore state of E289A-VCC illustrated the absence of density corresponding to the lower part of the transmembrane β-barrel scaffold (Figs. 7*B* and 8*B*). Despite the absence of density corresponding to the complete pore-forming scaffold, we observed strong lipid densities associated with the rim domain of this assembly state. This observation suggested that the membrane-proximal rim domain maintained tethering of the E289A-VCC oligomers on the membrane surface, as observed for WT-VCC (Fig. 8) (4).

### Mutation of E289A elevates the energy barrier associated with the pore-formation mechanism of VCC

**Figure 9:**
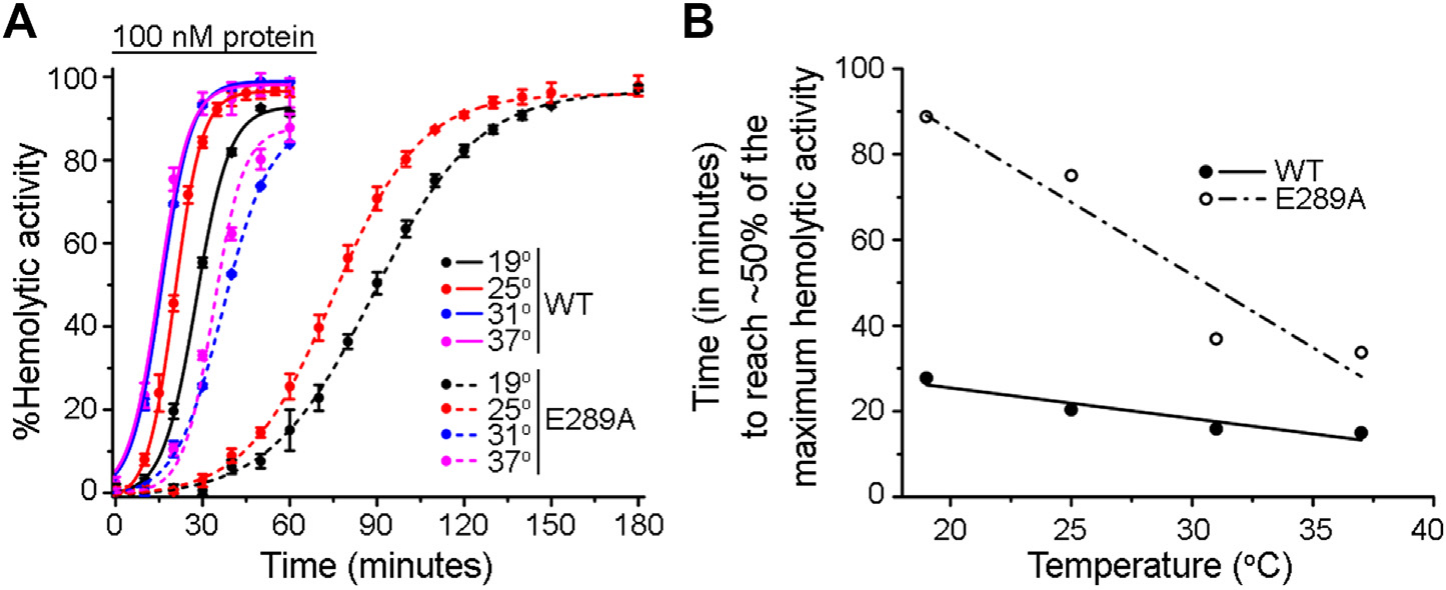
**E289A-VCC shows severely delayed kinetics of pore-forming hemolytic activity that can be rescued with elevated temperature conditions.***A*, pore-forming hemolytic activity of E289A-VCC (100 nM) was monitored at four different temperatures, by measuring the decrease in the turbidity of the erythrocyte suspension (corresponding to the absorbance at 650 nm). WT VCC (WT-VCC) was taken as control. Data shown here are the averages ± SDs from three independent treatments. Data were fitted to nonlinear curve-fitting model using dose-response function available within OriginPro software, and the fitted data are shown with *solid lines* (for WT-VCC) and *broken lines* (for E289A-VCC). *B*, time to reach ∼50% of maximum hemolytic activity (of E289A mutant and WT VCC; determined from the data shown in A) was plotted against temperature of the hemolytic activity assay condition. From the slopes of the linear fitting of the data, it is evident that the pore-forming efficacy of E289A-VCC is more severely dependent on temperature than that of WT VCC. VCC, *Vibrio cholerae* cytolysin.

E289A-VCC exhibited WT-like structural integrity and membrane interaction ability. Furthermore, it was capable of generating transmembrane β-barrel pore assembly, as documented in our cryo-EM study. Nevertheless, E289A mutant displayed severely compromised pore-forming hemolytic activity, as compared to that of WT-VCC. Toward exploring the mechanistic basis of such compromised pore-forming activity of E289A-VCC, we examined the possibility whether the mutation of E289A imposed a kinetic barrier on the pathway of functional pore formation by the membrane-bound toxin molecules. To explore this possibility, we measured the kinetics of hemolytic activity of E289A-VCC, along with those of WT-VCC as control, at four different temperatures (19 °C, 25 °C, 31 °C, and 37 °C). Results of this assay showed that at the lower temperatures of 19 °C and 25 °C, E289A-VCC displayed significantly delayed kinetics of hemolytic activity, as compared to those of WT toxin. Such a delayed kinetics of hemolytic activity of the mutant was rescued with the elevation of the temperature condition of the assay, particularly when examined at 31 °C and 37 °C (Fig. 9*A*). Furthermore, analyses of the hemolytic activity kinetics at varying temperature conditions (from the plot of ‘time to achieve ∼50% of the maximum hemolytic activity’ against the temperature of the assay) showed that the efficacy of pore-forming activity of E289A mutant was more severely dependent on temperature than that of WT-VCC (Fig. 9*B*). This observation clearly suggested that the energy barrier of the pore-formation mechanism increased when E289 residue was replaced with alanine.

## Discussion

Metamorphosis of the water-soluble β-PFT monomer into the transmembrane oligomeric β-barrel pore involves a number of prominent structural changes. Upon membrane-binding, toxin monomers assemble into the prepore oligomeric intermediates. Subsequently, the prestem motifs insert into the membrane lipid bilayer to create β-barrel stem of the pore. Structural rearrangement of the prestem motif is one of the most critical events in the β-PFT function. In the soluble monomeric state, the prestem motif remains packed against the central cytolysin domain. In the process of oligomeric pore formation in the membrane, prestem motif(s) of the toxin protomers detach from the cytolysin domain and establish new interactions with the β-strands of the neighboring prestem(s) toward forming the β-barrel scaffold (21, 29). It is needless to say that a precise design of the prestem motif is essential in the context of the β-PFT structure and function. Some of the earlier studies have shown that engineered disulphide bond–mediated locking and/or deletion of the pore-forming prestem motif block β-PFT pore-formation process and trap the toxins in the prepore-like states (24, 30, 31). Prestem motifs of the β-PFTs, in general, do not share any sequence similarity. Nevertheless, specific residue(s) within the pore-forming motif of the distinct β-PFTs are often found to play crucial roles. Therefore, it is important to examine the specific contributions of the prestem residues for deciphering the mechanism of action of the β-PFTs in exact detail.

In the present study, we found that the single point mutations of E289A and K304A in the prestem motif compromised the pore-forming efficacy of VCC (Figs. 1 and 2). Intrinsic tryptophan fluorescence–based study showed that the mutation of K304A resulted in a noticeable alteration in the structural integrity of the protein (Fig. 3). Aggregated assembly of K304A-VCC generated in the target membranes, as observed in the TEM imaging, also confirmed structural defect in this mutant protein (Fig. 6). Such defects in the overall structural integrity of the K304A mutant explain its compromised pore-forming activity.

Toward elucidating mechanistic basis of compromised pore-forming activity of E289A mutant, our study showed that E289A-VCC efficiently bound to the target membranes and formed SDS-stable oligomeric assembly with typical ring-like architecture, as observed with WT-VCC (Figs. 5 and 6). Furthermore, our cryo-EM data revealed that E289A-VCC was capable of forming WT-like transmembrane β-barrel pore assembly in the lipid bilayer (Figs. 7 and 8). Cryo-EM showed another oligomeric assembly state for the membrane-bound E289A-VCC, in which complete transmembrane β-barrel was not clearly resolved (Figs. 7 and 8). Similar assembly state with incomplete transmembrane β-barrel density was also documented in the previously reported cryo-EM study of WT-VCC (4). Documentation of this oligomeric assembly with incomplete β-barrel density raises the possibility that it might represent prepore-like intermediate state in the pathway of membrane pore formation by VCC. Indeed, formation of similar prepore oligomeric assembly states with incomplete β-barrel density have been documented previously in the cryo-EM studies on other structurally related β-PFTs (22, 23, 32). Based on the qualitative assessment of the cryo-EM data, relative abundance of this assembly state with incomplete β-barrel appeared to be noticeably higher in the case of E289A-VCC than that observed in the previously reported cryo-EM study of WT-VCC (4). Therefore, it is possible that the mutation of E289A in the prestem motif tends to arrest membrane-bound fraction of VCC in the prepore-like assembly state and interferes with its efficient transition into the functional transmembrane pore. We observed that the compromised pore-forming hemolytic activity of E289A-VCC could be rescued with the elevated temperature conditions of the assay (Fig. 9). Furthermore, with increasing temperature condition, the rate (in terms of ‘time to reach 50% of the maximum activity’) of pore-forming activity of the mutant was augmented more dramatically than that observed with WT VCC (Fig. 9). These results clearly reflected higher energy barrier associated with the pore-formation mechanism of E289A-VCC that, in turn, presumably resulted into drastically reduced kinetics of hemolytic activity of the mutant. Based on these observations, it appears that the mutation of E289A within the pore-forming motif of VCC tends to arrest membrane-bound toxin molecule in the prepore-like intermediate assembly state and compromises the efficacy to generate functional transmembrane β-barrel pore, presumably by raising the energy cost associated with the process. Notably, mutations of E289D and E289Q did not affect pore-forming activity of VCC to any noticeable extent. Therefore, it seems that the presence of a precise amino acid sidechain (E, D, or Q; and, not A) at the position 289 within the pore-forming motif of VCC is vital for its pore-formation mechanism.

**Figure 10:**
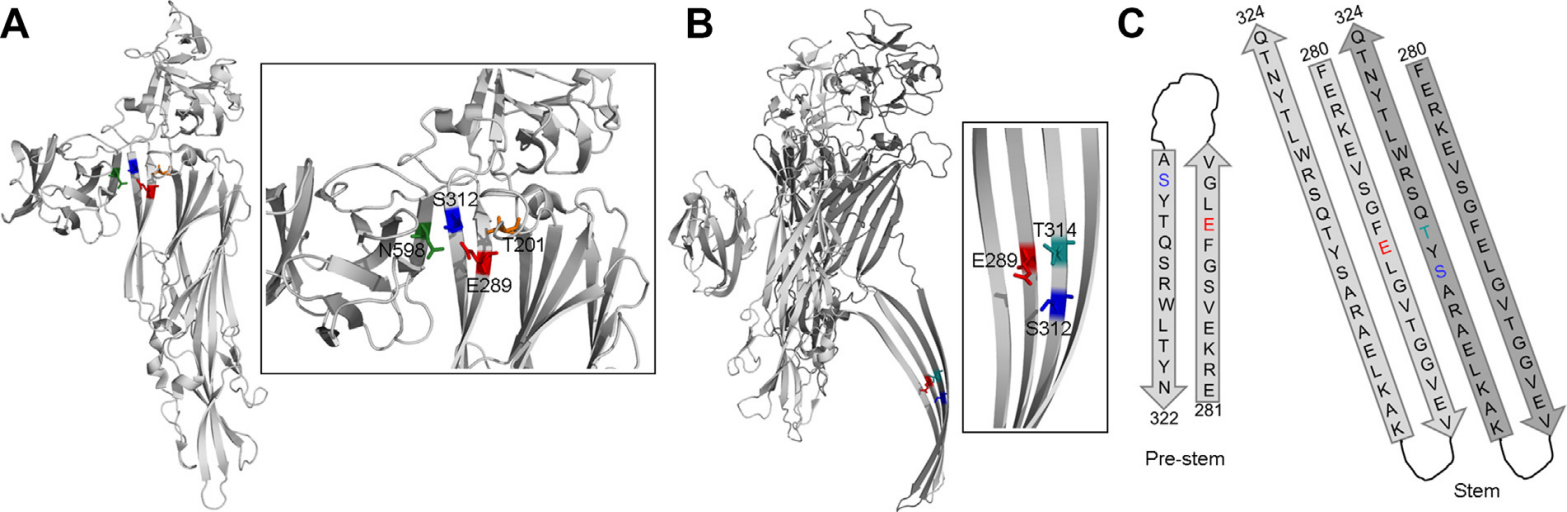
**Potential interaction partners of E289.***A*, proximity of E289 to S312, T201, and N598 in the monomeric state of VCC is shown. *B*, proximity of E289 to the residues S312 and T314 of the adjacent pore-forming motif contributed from the neighboring protomer in the transmembrane oligomeric β-barrel pore state of VCC is shown. *C*, arrangement of residues in the prestem (in the monomeric state) and stem (in the oligomeric β-barrel pore form) configuration of the pore-forming motif of VCC. For simplistic presentation, residues within the β-strand regions of the pore-forming motif are shown only. In the stem configuration, two neighboring β-strand pairs are shown in *light* and *dark gray* color. VCC, *Vibrio cholerae* cytolysin.

Efficient execution of the β-barrel pore-formation mechanism by VCC, or any β-PFT, involves stringently regulated structural rearrangement and membrane insertion of the pore-forming motif. It remains enigmatic how such processes are mediated in a well-orchestrated manner (14, 33). It is expected that the pore-forming motif residue(s) might play critical roles in regulating the efficacy of the pore-formation process. Consistent with such notion, it is evident from our study that the mutation of E289A in the pore-forming prestem motif of VCC tends to dampen conversion of the membrane-bound toxin molecules into the functional transmembrane pores. In the soluble monomeric state of VCC, three amino acid residues are found to have their sidechains located within a distance of less than 4 Å from that of E289. These residues are as follows: (i) S312 of the neighboring β-strand of the prestem motif (at a distance of ∼2.63 Å), (ii) N598 of the β-Prism domain (at a distance of ∼3.66 Å), and (iii) T201 of the cytolysin domain (at a distance of ∼3.96 Å)(Fig. 10*A*). Considering the sidechain properties of these residues, E289 (also D or Q at the position 289) can make potential hydrogen bonds and/or polar interactions with them in the monomeric state of VCC. Notably, neighboring residues of E289 change drastically in the transmembrane oligomeric pore state. S312, N598, and T201 are not in close proximity to E289 anymore within a toxin protomer. Instead, in the β-barrel scaffold of the toxin, E289 has only two proximal residues, S312 and T314 (residing within a distance of ∼3.7 Å), that are contributed by the pore-forming motif of the adjacent neighboring protomer (Fig. 10*B*). Based on these analyses, it appears that a number of polar contacts/hydrogen bond interactions involving the residue E289 get readjusted during the process of oligomeric β-barrel pore formation by VCC (Fig. 10*C*). Therefore, E289A mutation would be expected to jeopardize a coordinated reshuffling of these interactions. Readjustments of the polar contacts/interactions required for the functional β-barrel formation would be affected, presumably due to the absence of an appropriately charged/polar residue at the position of 289 (E, D, or Q). Accordingly, mutation of E289A would hamper appropriate structural rearrangement of the prestem motif and would dampen the efficacy of pore formation. This notion is supported by our data showing apparent high energy barrier and delayed kinetics of E289A mutant’s pore-forming activity, and it is further strengthened by our observation that the replacements of E289D and E289Q do not affect pore-forming activity of VCC. Future biochemical, structural, and in-silico studies will be required to provide more detailed insights regarding exact implications of the interaction network, involving the residue E289 and its interaction partners, in regulating coordinated transition of the membrane-bound VCC molecules into the functional pore state.

In summary, present study, for the first time, elucidates how a specific residue within the pore-forming motif of VCC regulates pore-formation mechanism by optimizing the energy barrier associated with the process and lays the ground work to explore in future whether similar regulatory roles are played by the pore-forming motif residues of the structurally related β-PFTs.

## Experimental procedures

### Ethics statement

Work with human blood was approved by the Institute Ethics Committee of Indian Institute of Science Education and Research, Mohali. Written informed consent was obtained from each donor. Studies reported in the article abide by the “Declaration of Helsinki” principles.

### Protein expression and purification

The recombinant WT VCC (WT-VCC) was generated following the method described in the previous studies (34). Briefly, the pET-14b vector harboring the nucleotide sequence of the precursor form pro-VCC was transformed into the *Escherichia coli* Origami B cells (Merck). Protein overexpression was induced with 1 mM IPTG (B. R. Biochem) at 30 °C for 3 to 4 h. Subsequently, pro-VCC was purified from the soluble fraction of the bacterial cell lysate using the Ni-NTA affinity chromatography (QIAGEN) followed by the Q Sepharose anion-exchange chromatography (Cytiva). Mature VCC was generated from pro-VCC by limited proteolysis with trypsin (protein:trypsin weight ratio of 2000:1) for 5 min at 25 °C. The mature VCC was further purified by Q Sepharose anion-exchange chromatography. The homogeneity of the protein was analyzed by SDS-PAGE/Coomassie staining. Protein concentration was estimated by measuring the absorbance at 280 nm, using the theoretical extinction coefficient calculated from the protein sequence.

Nucleotide constructs of all the single-point mutants used in this study were generated using the PCR-based method and were confirmed by DNA sequencing. The mutant proteins were expressed and purified following the same method described for WT-VCC.

### Intrinsic tryptophan fluorescence

Tryptophan fluorescence emission spectra of the VCC variants (1 μM) were recorded on a FluoraMax-4 (Horiba Scientific) spectrofluorimeter. The temperature was maintained at 25 °C using a Peltier-based temperature controller. Fluorescence emission spectra were collected over the range 310 to 400 nm upon excitation at 295 nm. Fluorescence emission spectra were corrected for the baseline by subtracting with the corresponding buffer (10 mM Tris–HCl, pH 8.0) spectra.

Thermal unfolding of the VCC variants was monitored by collecting the intrinsic tryptophan fluorescence spectra at different temperatures following the method described above. The ratio of the fluorescence emission intensities at 340 nm (wavelength region near the emission maximum of the native protein) and 355 nm (wavelength region near the emission maximum of the unfolded protein) (I_340_/I_355_) was determined at each temperature as an indicator for the overall tertiary structure of the protein.

### Assay of hemolytic activity

Hemolytic activity of the VCC variants against the human erythrocytes was performed following the methods described earlier (26, 34). Briefly, human erythrocytes were washed and resuspended in PBS (10 mM Na_2_HPO_4_, 1.8 mM KH_2_PO_4_, 137 mM NaCl, 2.7 mM KCl, pH 7.4). The amount of erythrocytes was adjusted in such a manner that either the absorbance at 650 nm was ∼0.9, or the complete lysis of the cells resulted in an absorbance of ∼0.9 at 415 nm, based on the experimental design of the hemolytic activity assay. For the assay of hemolytic activity, erythrocytes suspension was treated with 100 nM VCC variants for 1 h at 25 °C, unless mentioned otherwise. The extent of hemolysis was estimated spectrophotometrically by monitoring the decrease in the turbidity of the erythrocyte suspension (by measuring absorbance at 650 nm) or by measuring the release of hemoglobin (by measuring absorbance at 415 nm), upon cell lysis. Percent hemolytic activity was calculated by taking the buffer-treated cells as the negative control, and complete lysis of the cells upon treatment with WT-VCC corresponded to the 100% hemolytic activity.

### Flow cytometry–based assay of binding

Binding of the VCC variants with human erythrocytes was monitored by a flow cytometry–based assay described previously (26). Briefly, 10^6^ cells were treated with 75 nM proteins (WT-VCC or the mutants) in PBS. Then, the cells were stained with rabbit anti-VCC anti-serum (1:100, volume/volume) and FITC-labeled goat anti-rabbit secondary antibody (1:100, volume/volume; Sigma-Aldrich). All the incubations were performed for 30 min at 4 °C. Cells were analyzed on a FACSCalibur (BD Biosciences) flow cytometer. Cells treated with buffer and stained with the primary and secondary antibodies were taken as the negative control.

### Preparation of liposomes

Asolectin-cholesterol (1:1 weight ratio) liposomes were prepared following the method described earlier (34). Briefly, the lipids (5 mg) were dissolved in chloroform and transferred to a round-bottom flask. The organic solvent was evaporated, and the flask was vacuum-dried for 3 h to generate the lipid film on the flask surface. The lipid film was then hydrated with PBS (5 ml) for 3 h at 37 °C. The liposome suspension was extruded repeatedly by passing through a polycarbonate filter of 100 nm pore size (Avanti Polar Lipids).

### SPR-based assay

SPR-based assay was used to monitor the binding of the VCC variants with the membrane lipid bilayer of the Asolectin-cholesterol liposomes on a Biacore 3000 platform (GE Healthcare Life Sciences) following the method described earlier (26). The L1 sensor chip was conditioned with the Hepes buffer (20 mM Hepes, 150 mM NaCl, pH 7.5). Subsequently, the chip was prepared by injecting the liposome suspension (∼0.125 mg/ml) with a flow rate of 2 μl/min for 25 min and washed with one injection of 20 mM NaOH for 12 s with a flow rate of 100 μl/ml. To block the nonspecific binding, 0.1 mg/ml bovine serum albumin was injected for 3 min at a flow rate of 10 μl/min. For binding of the VCC variants, the protein was injected for 10 min with a flow rate of 5 μl/min, followed by washing with the Hepes buffer. After the completion of every experiment, the L1 chip was regenerated by removing the liposomes and bound proteins using one injection of 40 mM octyl β-D-glucopyranoside for 2 min at a flow rate of 10 μl/min. The corrected sensogram plots were generated using the BIAevaluation 4.1.1. software (GE Healthcare Life Sciences). SPR signal during the protein flow revealed the steady-state binding of the VCC variants, while the response units obtained during the buffer flow at the end of the protein injection were taken as the measure of the irreversibly bound protein on the liposome membranes.

### Pull down–based assay to monitor binding and oligomerization of the VCC variants in the liposomes

VCC variants (1 μM) were incubated with 130 μl Asolectin-cholesterol liposomes (from a stock preparation of ∼1 mg/ml) in a reaction volume of 1 ml in PBS for 1 h at 25 °C. The reaction mixture was subjected to ultracentrifugation at 105000*g* for 30 min. After separating the supernatant, the pellet fraction was washed twice with PBS and then resuspended in 1 ml PBS. Equal volumes (20 μl) of the supernatant and the pellet fractions were analyzed by SDS-PAGE/Coomassie staining after boiling in the presence of the SDS-PAGE sample buffer.

For monitoring the SDS-stable oligomer formation by the VCC variants in the liposomes, proteins (1 μM) were incubated with 130 μl Asolectin-cholesterol liposomes (from a stock preparation of ∼1 mg/ml) in 1 ml reaction volume in PBS for 1 h at 25 °C. The reaction mixture was then subjected to ultracentrifugation at 105000*g* for 30 min. The pellet fraction was collected, washed twice with PBS, resuspended in 50 μl SDS-PAGE sample buffer, and was then divided into two equal parts. One part was boiled for 15 min, and the other part was incubated at 25 °C for 15 min (unboiled fraction). The samples were then analyzed by SDS-PAGE/Coomassie staining.

### Pull down–based assay to detect the SDS-stable oligomer formation by the VCC variants in the erythrocytes membrane ghost

Membrane ghosts of the human erythrocytes were prepared following the method described earlier (34). Briefly, 3 ml human blood was washed with PBS and subsequently resuspended in an ice-cold lysis buffer (4 mM Na_2_HPO_4_.2H_2_O, 1 mM NaH_2_PO_4_, 1 mM EDTA, pH 7.5). The cell suspension was incubated at 4 °C for 30 min and then centrifuged at 15000*g* for 30 min. The pellet fraction from the lysed erythrocytes was collected and washed multiple times with the lysis buffer until the fraction was completely devoid of any unlysed cells. The pellet fraction containing the erythrocytes membrane ghost was then resuspended in a 10 mM sodium phosphate buffer, containing 5 mM MgCl_2_ (pH 7.5), and was incubated at 37 °C for 40 min. After the incubation, the suspension was centrifuged at 15000*g* for 30 min, and the pellet was resuspended in 5 ml 10 mM sodium phosphate buffer, containing 5 mM MgCl_2_ (pH 7.5), and mixed thoroughly. The protein concentration in the human erythrocytes membrane ghost preparation was estimated by a standard Bradford assay and was found to be ∼1.4 mg/ml.

Fifty microliters of the erythrocytes membrane ghosts were incubated with 100 nM VCC variants in a reaction volume of 1 ml in PBS for 1 h at 25 °C. The reaction mixture was then subjected to ultracentrifugation at 105000*g* for 30 min. The pellet was collected, washed twice with PBS, resuspended in 50 μl SDS-PAGE sample buffer, and was then divided into two equal parts. One part was boiled for 15 min, and the other part was incubated at 25 °C for 15 min (unboiled fraction). Samples were then subjected to SDS-PAGE followed by immunoblotting (with rabbit anti-VCC anti-serum as primary antibody and HRP-conjugated anti-rabbit secondary antibody (Sigma-Aldrich)). Immunoblot was developed with the Clarity Western ECL Substrate (Bio-Rad), and images were acquired on an ImageQuant LAS4000 (GE Healthcare Life Sciences).

### Negative staining and TEM

Negative stain TEM was used to visualize the assembly states of the VCC variants upon association with the membrane lipid bilayer of the Asolectin-cholesterol liposomes and human erythrocytes membrane ghost. VCC variants (∼6 μM) were incubated with ∼1 mg of Asolectin-cholesterol liposomes in a reaction volume of 2 ml in 10 mM Tris–HCl buffer (pH 8.0) or with 25 μl of erythrocytes membrane ghosts in a reaction volume of 1 ml in 10 mM Tris–HCl buffer (pH 8.0), for 1 h at 25 °C. The reaction mixtures were subjected to ultracentrifugation at 105000*g* for 30 min at 4 °C. The pellet fractions were collected, washed twice, and resuspended in 200 μl of 10 mM Tris–HCl buffer (pH 8.0) containing 150 mM NaCl.

For the negative staining and TEM imaging, 400 mesh copper grids with continuous carbon support (Agar Scientific) were plasma cleaned using a Solarus II plasma cleaner (Gatan Inc) for 30 s. Five microliters of the resuspended pellet fraction (as prepared above) was applied to the plasma cleaned grid and incubated for 1 min. The excess amount of the sample was then gently blotted off and the sample was stained with 2% uranyl acetate. TEM images were recorded using a JEM-F200 electron microscope (Jeol Inc) operated at 200 kV. The images were recorded using a Gatan OneView camera (Gatan Inc). The micrographs were recorded at a nominal magnification of 600,00× and 800,00× for the samples with the liposomes and erythrocyte ghosts, respectively, which resulted in object level pixel sizes of 1.65 Å and 2.2 Å, respectively. Images were collected with a nominal defocus of -2 μm to -3 μm and the cumulative fluence for each image was limited to ∼120 electrons/pixel.

### cryo-EM sample preparation and data collection

Quantifoil R1.2/1.3300 holey carbon Cu grids were hydrophilized in GloQube (Quorum Technologies Ltd) glow discharge system, for 90 s at 20 mA prior to sample application. Subsequently, 3 μl of liposome-bound E289A-VCC sample (prepared as described above for the negative staining-TEM) was added to freshly glow discharged cryo-EM grids and incubated for 10 s. The surplus sample was blotted for 8.5 s at 100% humidity and plunge-frozen into liquid ethane cooled by ambient liquid nitrogen, using a FEI Mark IV vitrobot. Cryo-EM movies were acquired at cryogenic condition in Thermo Scientific 200 kV Talos Arctica Transmission Electron Microscope equipped with K2 Summit Direct Electron Detector (Gatan Inc). Image acquisition was done using LatitudeS (35) automatic data collection software (Gatan Inc) at a nominal magnification of 540,00× and a pixel size of 0.92 Å at sample level. The specimen was subjected to a total electron dose of about 40 e^-^/Å^2^ between the defocus range of -0.75 μm and -2.5 μm at a calibrated dose of about 2 e^-^/Å^2^ per frame. Data were recorded for 8 s for a total of 20 frames.

### Cryo-EM data processing and 3D reconstruction

Two thousand multi-frame movies were imported into RELION 3.1 (36) and beam-induced motion correction was performed over frames 2 to 17 using RELION implementation of MotionCor2 (37). Following this, motion-corrected micrographs were screened in cisTEM (38) and only the micrographs that showed best signal to noise ratio were retained for single particle analysis. The contrast transfer function was estimated with CTFFIND 4.1.13 (39). At first, a subset of 100 micrographs was used to manually pick particles in RELION 3.1, and reference-free 2D class averages were calculated. The 2D class averages with the best signal to noise ratio were selected as template for autopicking 1300 final micrographs. Two lakhs thirty four thousand one hundred seventy-six particles were obtained with automated picking, which were further curated by performing multiple rounds of 2D classification. Around 148,249 best particles were subjected to 3D classification into 10 classes with C7 symmetry (Fig. S2). Two high-resolution 3D models with distinct lipid environment were selected for autorefinement. Prior to refinement, a soft mask was generated against both the 3D classes in RELION 3.1. For each particle subset, contrast transfer function refinement was performed with the correction of anisotropic magnification, beamtilt, per-particle defocus, and per-particle astigmatism. Finally, the particles were polished and the “shiny” particles were imported into cryoSPARC 3.2 (40). A single round of homogeneous refinement followed by local refinement using C1 symmetry was performed in cryoSPARC 3.2 to yield final reconstructions at 4.7 Å and 6.4 Å (FSC 0.143) with 42,124 and 18,486 particles, respectively.

### Real-space refinement and structural analyses

Map to model correlation of the relatively higher resolution map of the complete transmembrane β-barrel pore-state of E289A-VCC was performed with a real-space–refined atomic model derived from the PDB (Protein Data Bank) ID 3O44. Initially, rigid-body fitting of PDB ID 3O44 into the 4.7 Å map of E289A-VCC was performed in UCSF Chimera (41). Seven strain-related mutations as per the UniProt ID A0A0H6SZL4, along with the E289A mutation, were incorporated in the PDB ID 3O44 using Coot (42). The fitted 3O44 (PDB ID) model with additional mutations was next considered as an initial model for phenix.dock_in_map in Phenix (43). Following this, real-space refinement of the model was performed using phenix.real_space_refine (Table S1). EMRinger score of the cryo-EM map was calculated in Phenix (Table S1). For the comparatively lower resolution map of the E289A-VCC oligomeric state with partial and distinctly short β-barrel density, rigid-body docking was done using the real-space–refined atomic model corresponding to the complete pore state of E289A-VCC. All 3D structural visualizations were performed in UCSF Chimera (41) and UCSF ChimeraX (44).

### Visualization of the protein structural models

Protein structural models were visualized with PyMOL (Schrodinger) unless mentioned otherwise. Structural models were generated using the protein structural coordinates of the monomeric state and the oligomeric pore form of VCC retrieved from the protein data bank (PDB)(PDB IDs: 1XEZ corresponding to the monomeric Pro-VCC and 3O44 for the oligomeric pore assembly of VCC).

## Data availability

The EM density maps have been deposited to the Electron Microscopy Data Bank (EMDB) with the accession codes EMD-33215 (for E289A-VCC mutant with complete transmembrane β-barrel) and EMD-33219 (for E289A-VCC mutant with partial transmembrane β-barrel). The coordinates for the atomic model obtained using the cryo-EM map of E289A-VCC mutant with complete transmembrane β-barrel have been deposited in RCSB PDB with the PDB ID: 7YL9.

All other data are contained within the article.

## Supporting information

This article contains supporting information (Figs. S1–S3, and Table S1).

## Conflict of interest

The authors declare that they have no conflicts of interest with the contents of this article.

## Supporting information

SupplementaryInformation
