# Supplementary material for: Glu289 residue in the pore-forming motif of Vibrio cholerae cytolysin is important for efficient β-barrel pore formation
Source: J Biol Chem. 2022 Aug 31;298(10):102441. doi: 10.1016/j.jbc.2022.102441 (PMC9520032; doi:10.1016/j.jbc.2022.102441)
Supplement: SupplementaryInformation [file mmc1.pdf]

Glu289 residue in the pore-forming motif of *Vibrio cholerae* cytolysin is important for efficient β-barrel pore formation

Anish Kumar Mondal[1], Nayanika Sengupta[2], Mahendra Singh[1], Rupam Biswas[2], Kusum Lata[1], Indrajit Lahiri[1], Somnath Dutta[2] and Kausik Chattopadhyay[1,*]

[1]Department of Biological Sciences, Indian Institute of Science Education and Research Mohali, Sector 81, S. A. S. Nagar, Manauli, Punjab 140306, India

[2]Molecular Biophysics Unit, Indian Institute of Science, Bangalore 560012, India

*Corresponding Author: Dr. Kausik Chattopadhyay, Department of Biological Sciences, Indian Institute of Science Education and Research Mohali, Sector 81, SAS Nagar, Manauli, Mohali, Punjab 140306, India. Tel: 91-0172-2293147; Fax: 91-0172-2240124.
E-mail: kausik@iisermohali.ac.in; ORCID: 0000-0001-8529-9475

Running title: Pre-stem residue regulates VCC pore-formation

Keywords: bacterial toxin; pore-forming toxin; *Vibrio cholerae* cytolysin; membrane; transmembrane domain; oligomerization; membrane pore; pre-pore; protein structure; cryo-EM.

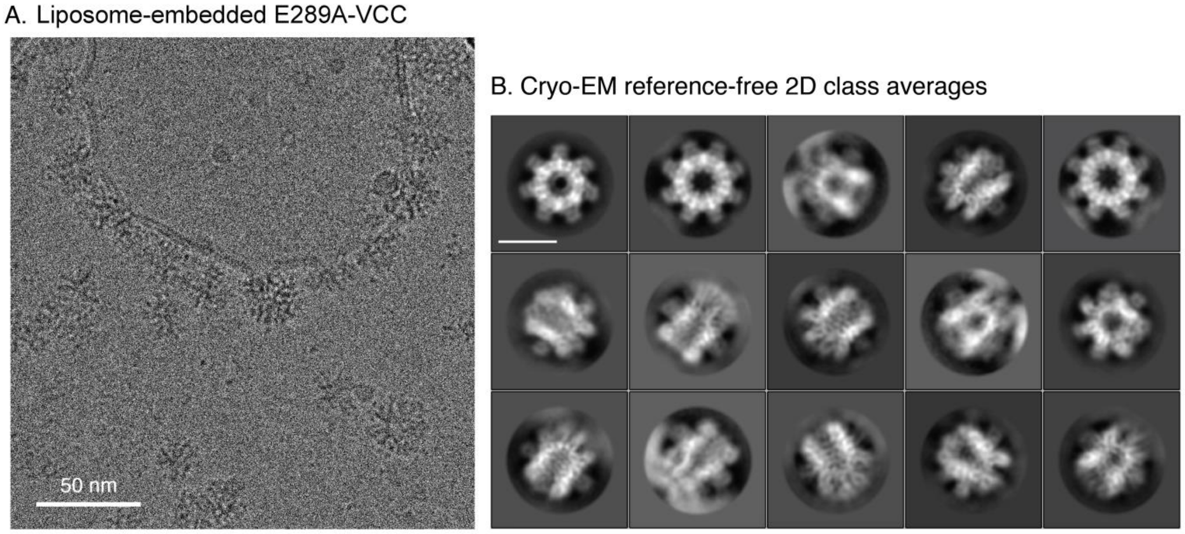

Fig. S1. (A) Cryo-EM raw micrograph showing the particle distribution of liposome-bound oligomeric assembly states of E289A-VCC in vitreous ice. (B) Extended 2D class averages represent the different orientations of membrane-bound E289A-VCC. Scale bar indicates 100 Å.

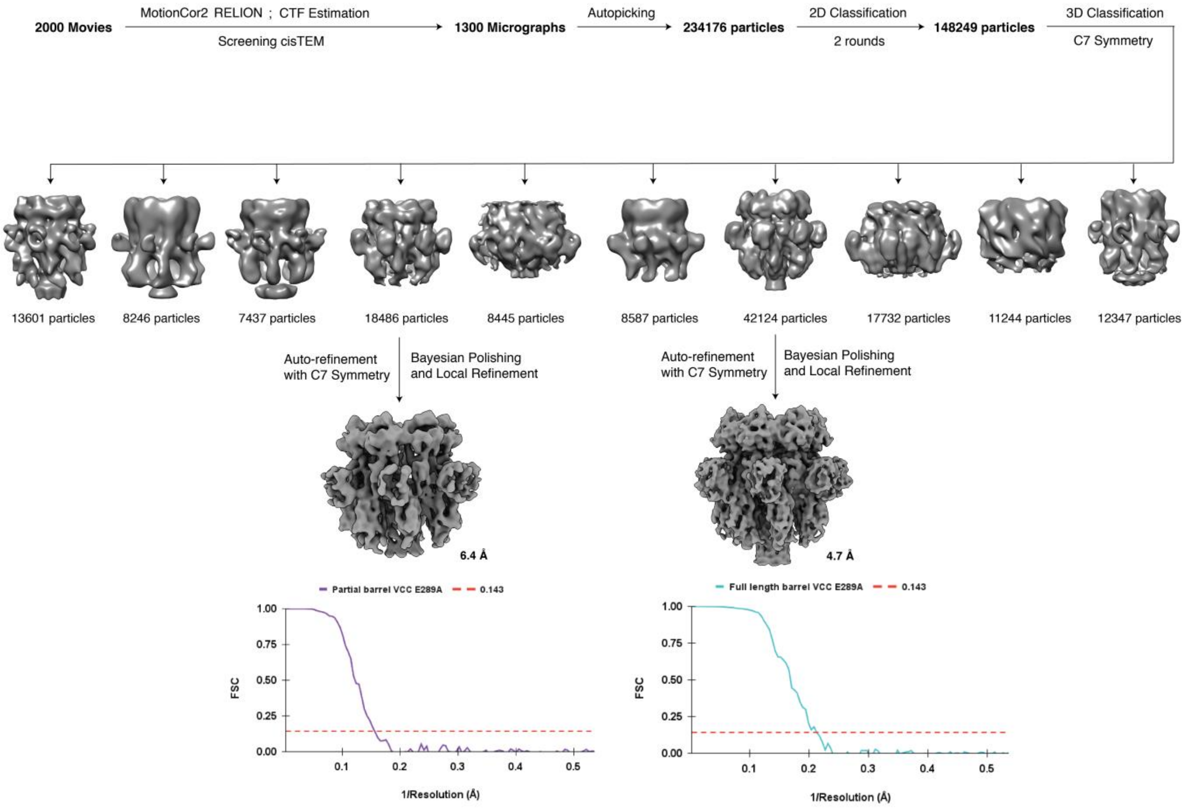

Fig. S2. Cryo-EM data processing pipeline of liposome-bound E298A-VCC.

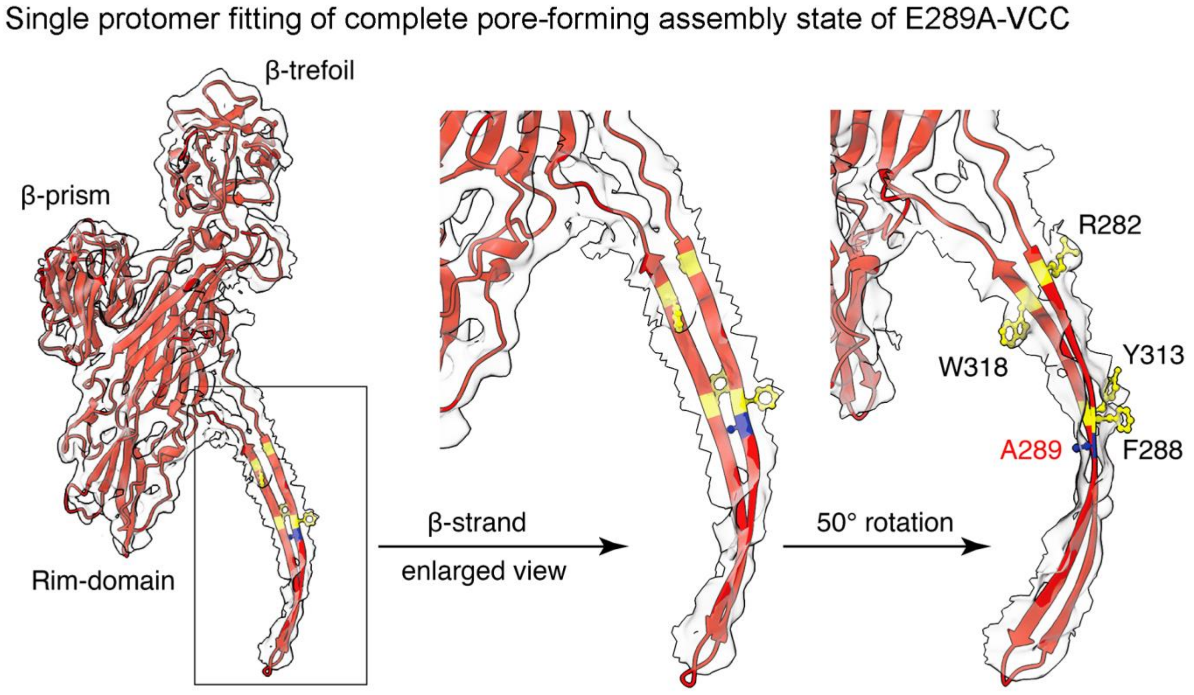

Fig. S3. Fitting of the secondary structural elements into a single protomer obtained from single-particle reconstruction of E289A-VCC shows a good agreement with the atomic model (PDB ID 7YL9). Enlarged view of a single transmembrane antiparallel β-strand shows the side chain fitting of bulky amino acid residues such as W318, R282, Y313, F288. The position of E289 in the atomic model is represented as blue ball-and-stick and the absence of the side chain density in the equivalent position of cryo-EM structure of E289A mutant is observed.

Table S1: Cryo-EM data collection, processing and refinement statistics.

	**Complete transmembrane pore state of E289A-VCC (EMD-33215) (PDB: 7YL9)**	**E289A–VCC oligomer with partial transmembrane β-barrel (EMD-33219)**
**Data collection and processing**		
Microscope model	TALOS ARCTICA	TALOS ARCTICA
Magnification	54000x	54000x
Pixel size (Å)	0.92	0.92
Voltage (kV)	200	200
Electron dose per frame ($e^-/Å^2$)	2	2
Number of frames	20	20
Defocus range (µm)	-0.75 to -2.50	-0.75 to -2.50
Initial particle images (no.)	234176	234176
Final particle images (no.)	42124	18486
Symmetry imposed	C7	C7
Map resolution (Å)	4.7	6.4
FSC threshold	0.143	0.143
Map sharpening B-factor ($Å^2$)	-276.9	-476.1
**Model Refinement Statistics**		
Initial model PDB ID	3O44	-
Model resolution (Å)	4.7	-
FSC threshold	0.143	-
**Composition (no.)**		-
Chains	7	-
Non-hydrogen atoms	31115	-
Protein residues	4067	-
Ligands	0	-
**Bonds (RMSD)**		-
Length (Å)	0.005 (0)	-
Angles (°)	0.766 (12)	-
Molprobity score	2.4	-

Clash score	28.88	-
**Ramachandran Plot (%)**		-
Favoured	92.97	-
Allowed	6.79	-
Outliers	0.25	-
**Rama-Z (Ramachandran plot Z-score, RMSD)**		-
Whole (N=4053)	1.95 (0.13)	-
Helix (N=84)	2.85 (0.48)	-
Sheet (N=1484)	0.80 (0.13)	-
Loop (N=2485)	1.58 (0.12)	-
Rotamer outliers (%)	0	-
Cβ outliers (%)	0	-
CaBLAM outliers (%)	2.7	-
**Peptide plane (%)**		-
Cis proline/general	0.0/0.0	-
Twisted proline/general	0.0/0.0	-
EMRinger score	1.07	-
